# Modeling of Longitudinal Changes in Left Ventricular Dimensions among Female Adolescent Runners

**DOI:** 10.1371/journal.pone.0140573

**Published:** 2015-10-15

**Authors:** Norimitsu Kinoshita, Fuminori Katsukawa, Hajime Yamazaki

**Affiliations:** 1 Faculty of Sports and Health Studies, Hosei University, Machida, Tokyo, Japan; 2 Sports Medicine Research Center, Keio University, Yokohama, Kanagawa, Japan; Victoria University, AUSTRALIA

## Abstract

**Purpose:**

Left ventricular (LV) enlargement has been linked to sudden cardiac death among young athletes. This study aimed to model the effect of long-term incessant endurance training on LV dimensions in female adolescent runners.

**Methods:**

Japanese female adolescent competitive distance runners (*n* = 36, age: 15 years, height: 158.1 ± 4.6 cm, weight: 44.7 ± 6.1 kg, percent body fat: 17.0 ± 5.2%) underwent echocardiography and underwater weighing every 6 months for 3 years. Since the measurement occasions varied across subjects, multilevel analysis was used for curvilinear modeling of changes in running performance (velocities in 1500 m and 3000 m track race), maximal oxygen uptake (VO_2_max), body composition, and LV dimensions.

**Results:**

Initially, LV end-diastolic dimension (LVEDd) and LV mass were 47.0 ± 3.0 mm and 122.6 ± 15.7 g, respectively. Running performance and VO_2_max improved along with the training duration. The trends of changes in fat-free mass (FFM) and LVEDd were similarly best described by quadratic polynomials. LVEDd did not change over time in the model including FFM as a covariate. Increases in LV wall thicknesses were minimal and independent of FFM. LV mass increased according to a quadratic polynomial trend even after adjusting for FFM.

**Conclusions:**

FFM was an important factor determining changes in LVEDd and LV mass. Although running performance and VO_2_max were improved by continued endurance training, further LV cavity enlargement hardly occurred beyond FFM gain in these adolescent female runners, who already demonstrated a large LVEDd.

## Introduction

Many cross-sectional studies have indicated that young trained endurance athletes have larger left ventricles than sedentary controls [1–5]. This enlargement, referred to as “athlete’s heart,” has been regarded as a consequence of physiologic adaptation of the left ventricles to realize larger cardiac output and sustain the higher oxygen demand of exercised skeletal muscles [1][2]. This training-induced adaptation has been reported to occur in female athletes [3] or adolescent populations [4].

The LV end-diastolic dimension (LVEDd) observed in highly trained endurance athletes is likely to exceed the normal limit [6], and differentiation from dilated cardiomyopathy can be a great challenge to clinicians in terms of preventing sudden cardiac death in young athletes [7]. Examination of prolonged exposure to exercise training is necessary to determine normative change [8]. Several longitudinal and prospective studies also demonstrated endurance exercise training-induced left ventricular (LV) enlargement in formerly untrained or sedentary young individuals [9–15]. However, long-term observational evidence is scarce for female adolescent distance runners.

In a longitudinal study, change in measured values has been evaluated by a pretest-posttest analysis which has been criticized in that it may cause erroneous results if the size of an increment is relatively small compared to measurement error [16]. Compared to this two-wave design, studies of multiple waves of data are beneficial to illustrate the shape of change over time and therefore minimize the possibility of confounding true change with measurement error [16]. For studies of this design, therefore, it is preferable to use multilevel analysis, which does not require independence of error and is advantageous over traditional statistical methods such as paired *t* tests or repeated-measure one-way analysis of variance (ANOVA) [17].

From these points of view, we conducted a prospective and longitudinal study with multiple measurement occasions on young, trained female runners with highly homogeneous backgrounds and analyzed the longitudinal repeated data by multilevel analysis. The purpose of the study was to elucidate the effect of long-term intensive endurance training on the left ventricle by modeling trajectories of longitudinal changes in LV dimensions among already trained adolescent female runners.

## Methods

### Subjects

This prospective study originated from the medical support program for competitive runners of the Sports Medicine Research Center, Keio University, started in 2001. The subjects were Japanese female middle- or long-distance (800 to 5000 m) runners belonging to the same competitive distance running team of a girls’ high school. Subjects were recruited every year as 15-year-old freshmen and regularly evaluated for 3 years until they finished high school. A total of 51 runners were recruited into the study. Five runners who quit the team after less than 2 years and 10 runners who changed their disciplines to a shorter distance (e.g., 400 m) or the walk were excluded from the analysis. Ultimately, 36 runners were included in the study.

The team consistently ranked first or second in the prefectural championship road relay race during the study period and often in the top level of the relevant national championship. Most of the subjects already had about 3 years of competitive middle- or long-distance running experience in their junior high schools prior to the study and were recruited to the high school team on the basis of those achievements. The subjects included many runners who set new prefectural high school records in 800–5000-m distances during the observational period or who became professional runners after they graduated from high school. Some of the subjects lived in the same team dormitory, and in general, their daily lifestyle and diet were strictly controlled, as was the discipline of physical training by the same coaches. Training consisted of 2 sessions almost every day: 1 hour in the early morning before school and 2 or 3 hours in the evening after school. Most of the training was endurance-type: jogging, tempo running, or high-intensity interval training. Resistance and agility training were also included to a lesser extent. Each runner recorded details of the training and diet every day, and roughly estimated running mileage was at most about 400 km per month.

All subjects included in this analysis were judged to be free of structural cardiovascular disease on the basis of medical history at initial evaluation, mandatory standard 12-lead electrocardiography performed in the high school, and consecutive echocardiography.

### Ethics Statement

This study was approved by the institutional ethical board of the Sports Medicine Research Center, Keio University (No. 2013–01). All procedures of the study were conformed to the latest revision of the Declaration of Helsinki. Written consent forms signed by subjects and their parents were obtained.

### Echocardiography

Doppler and 2-D echocardiography were performed in conjunction with M-mode imaging using a SONOS 5500 imaging system (Philips Healthcare, Bothell, WA, USA) equipped with a 2–4-MHz broadband linear transducer (S4 probe). Measurements of LV dimensions were obtained from M-mode recordings of the 2-D parasternal short axis view of the left ventricle at the level of the tips of the mitral leaflets, recorded at 100 mm/s under the guidance of direct anatomical visualization. Although the measurements were conducted essentially according to the recommendations of the American Society of Echocardiography [18], special care was taken to identify the LV endocardial border throughout the cardiac cycle. In addition, we used magnified images of the septal and posterior walls of the left ventricle to visualize structures in detail while avoiding the inclusion of trabeculations or other structures superimposed on the LV wall. To maintain the consistency of the quality of serial measurements, all measurements were performed by the same person (NK), and special care was taken to set the same depth of the 2-D parasternal short axis view for M-mode recordings on every occasion for each subject. LV mass was calculated using the formula of Devereux et al. [19]. Blood pressure was measured before echocardiography with subjects in a sitting position, and resting heart rate (HR) was recorded at the measurement of LV dimensions during echocardiography

### Anthropometric measurements

Body composition was assessed by underwater weighing (AD-6204, A & D, Tokyo, Japan) on the same day as echocardiography, in accordance with the manufacturer’s instructions. Body density was computed and used to predict percent body fat using the equation of Brozek et al. [20]. Fat mass and fat-free mass (FFM) were calculated based on percent body fat and body weight. Body surface area (BSA) was calculated according to the formula of DuBois and DuBois [21].

### Maximal oxygen uptake (VO_2_max)

Maximal exercise test was performed using a treadmill on the day of or one day after echocardiography, only if the subjects were judged to be able to run with maximum exertion. Therefore, VO_2_max data were not necessarily available for all echocardiography and underwater weighing sessions. The average number of VO_2_max tests was 2.7 ± 1.3/subject (range: 1–6). The protocol for determining VO_2_max consisted of a series of 1-min stages, starting at 9–10 km/h on a 1–3% grade. The starting speed and slope were determined on the basis of each subject’s running capability, estimated by their performance records, so that the subjects could continue the test for 10 minutes [22]. The speed was increased by 1 km/h at each stage until volitional exhaustion, with the slope fixed to the initial setting. All subjects were given frequent, strong verbal encouragement to go all out until they could go no more, at which point the test was ended. A harness that hung from the ceiling and was loosely connected to the subjects during tests guaranteed their safety by preventing them from falling on the treadmill. During the exercise, HR was monitored using the bipolar CM5 lead of an electrocardiograph and recorded continuously on a Life Scope 6 monitor (Nihon Kohden, Japan). Oxygen uptake and carbon dioxide production were measured using a breath-by-breath Quark *b* ^2^ or Quark CPET (Cosmed, Rome, Italy) metabolic cart. The gas analyzers were calibrated before and after each test with air and a commercial gas of known composition, as described in the manual. VO_2_max (averaged over 3 consecutive 10-s intervals) was defined as the point at which oxygen uptake plateaued, despite increasing work rate (leveling-off criterion), with a respiratory quotient > 1.10 [23].

### Statistical analysis

#### Descriptive characteristics

Means and standard deviations (SDs) of variables were calculated on each occasion without considering the longitudinal repeated data structure. Paired *t* tests were conducted using the first and last tests of each subject during the 3-year period to illustrate the magnitudes of the changes in body size/composition variables, resting HR, and blood pressure. The mean duration between the first and last data collection was 680 ± 180 days (range: 351–894 days) for body size/composition variables and 653 ± 160 days (range: 351–883 days) for resting HR and systolic and diastolic blood pressure.

#### Performance change

Besides everyday training, the subjects regularly participated in track and field competitions. All 36 subjects participated in 1500 m races, while 33 also participated in 3000 m races. The mean number of times the subjects participated in the 1500 m race was 12.1 ± 5.6 and in the 3000 m race was 16.2 ± 5.1, over the 3-year follow-up period. To demonstrate the effect of exercise training, we analyzed the change in subjects’ running performance using the official records of those track races—a total of 453 and 590 records for the 1500 m and 3000 m races for the 36 and 33 subjects, respectively. Mean running velocities (m/min) for each distance (V_1500_ and V_3000_, respectively) were calculated, and days from March 1st of the first year (DAYS) of the observation, when the subjects joined the team, were counted for every race occasion of each subject.

We attempted to construct polynomial growth curve models to describe the changes to V_1500_ and V_3000_ along with DAYS using multilevel analysis, because occasions of competitions varied by subject and the number of available records was not the same for all subjects. Multilevel analysis is advantageous for this type of longitudinal data, which could be viewed as two-level data with repeated measurements nested within individuals [24]. We evaluated 3 random intercept fixed-slope models: linear (fixed-effect = DAYS), quadratic (fixed-effects = DAYS and DAYS^2^), and cubic (fixed-effects = DAYS, DAYS^2^, and DAYS^3^), where a variance component was assumed for covariance structure of the random component (intercept). The maximum likelihood method was used to estimate the regression coefficients and the intercept and slope variances. Chi-square tests were performed to compare the 3 models using the change in the values of minus twice the log-likelihood (*–2LL*) against the change in the number of parameters of each model.

#### Modeling of the changes in LV dimensions

The first echocardiography measurement and underwater weighing were performed in the spring season when subjects had joined the team but not yet started training. The measurements were repeated every 6 months, i.e., there were 2 measurement occasions per year (first, March to April and second, September) and a total of 6 occasions over 3 years. Each occasion of measurement (T_measure_) was numbered 0, 1, 2, 3, 4, and 5 instead of 1 to 6 for statistical analysis, so that ‘zero’ (intercept) was part of the range of possible values for the longitudinal data. One hundred thirty-one data sets of body composition and echocardiography data were collected from the 36 subjects. The numbers of measurements were not the same for all subjects. The subjects often missed measurement occasions because of injury or participation in competitions or training camps on the examination day. Missing echocardiography or body size/composition data were assumed to be missing completely at random, with missingness independent of all other variables. Among the 36 subjects, 7 were measured on 2 occasions, 12 were measured 3 occasions, 7 were measured 4 occasions, 7 were measured 5 occasions, and 3 were measured all 6 occasions. As a result, the number of subjects differed on each measurement occasion, implying that the data was not time-balanced according to the occasions and the available number of measurements varied by subjects. Therefore, we performed multilevel analysis to model the time trend of changes in LV dimensions, because multilevel analysis does not require time-structured balanced data [16]. The longitudinal repeated measures of LV dimensions were regarded as a two-level model with repeated measurements nested within individuals as in the analysis of performance change, with the series of repeated measures at the lower level (Level-1) and individual subjects at the higher level (Level-2).

First, we tested a no-predictor model (unconditional mean model) to partition the variance in dependent variables into within- and between-subject components. A variance component was selected for covariance structure of the random component (intercept). The intraclass correlation (ICC) was calculated by dividing between-subject variance by total variance. Given subjects’ similar background, the quantity and quality of physical training among subjects could be regarded as relatively consistent during follow-up. For the next step, therefore, we assumed that the effect of training on LV dimensions did not differ by subject, and a random intercept and fixed-slope model was hypothesized to define the longitudinal changes in LV dimensions. In this analysis, a variance component was selected for Level-2 covariance structure, and Level-1 covariance structure was defined to be a homogeneous first-order autoregressive structure where the residual errors were assumed to be correlated from occasion to occasion within subjects but independent across subjects. We analyzed both linear and quadratic models where predictor variables were T_measure_ only (model-1) and T_measure_ and square T_measure_ (model-2), respectively. We also applied the same procedure to model the longitudinal change in VO_2_max, FFM, and fat mass. Regarding the VO_2_max, we tested models additionally using LVEDd or LVM as a covariate. Finally, we tested FFM-adjusted models for VO_2_max and LV dimensions. Again, both linear and quadratic models were tested (model-3 and -4, respectively). Chi-square tests were performed to compare the models using the change in the values of –*2LL* against the change of the number of parameters of each model. All statistical analyses were performed with SPSS^®^ 15.0J for Windows (IBM, Japan), with multilevel analysis implemented through the Linear Mixed Models procedure. *P* < 0.05 was considered statistically significant.

## Results

### Descriptive characteristics

Cross-sectional means and SDs of variables calculated on each occasion without considering the data structure of repeated measures are shown in Table 1. Paired *t* tests between the first and last measurements for individual runners revealed a small but significant increase in body mass (45.3 vs. 46.9 kg, *p* < 0.001), while fat mass decreased and FFM increased significantly (7.4 vs. 6.6 kg, *p* < 0.001; 37.9 vs. 40.3 kg, *p* < 0.001, respectively). Resting HR significantly decreased, but systolic/diastolic blood pressures did not change (58.1 vs. 50.7 bpm, *p* = 0.01; 103.8 vs. 98.8 mmHg, *p* = 0.37; 62.6 vs. 63.0 mmHg, *p* = 0.38, respectively).

**Table 1:** Means and standard deviations of LV dimensions and other variables for each occasion of measurement.

	Occasions of measurement (T_measure_)					
	0	1	2	3	4	5
Number of subjects	26	17	34	13	29	12
Height (cm)	158.1 ± 4.6	159.6 ± 6.0	159.4 ± 5.0	159.8 ± 5.6	160.5 ± 5.0	160.5 ± 5.6
Body mass (kg)	44.7 ± 6.1	46.4 ± 5.6	46.5 ± 5.1	47.1 ± 4.8	47.1 ± 4.3	48.8 ± 3.9
Fat mass (kg)	7.8 ± 3.2	6.8 ± 2.3	6.6 ± 2.0	6.7 ± 1.7	6.6 ± 2.1	7.2 ± 2.7
FFM (kg)	37.0 ± 4.3	39.6 ± 4.7	39.9 ± 4.2	40.4 ± 4.5	40.5 ± 3.7	41.6 ± 3.6
Percent body fat (%)	17.0 ± 5.2	14.6 ± 3.9	14.0 ± 3.6	14.2 ± 3.4	14.0 ± 3.8	14.6 ± 4.8
Resting heart rate (bpm)	59 ± 11	56 ± 9	50 ± 8	57 ± 9	50 ± 8	53 ± 12
Systolic blood pressure (mmHg)	105 ± 10	104 ± 7	97 ± 9	101 ± 8	101 ± 9	100 ± 8
Diastolic blood pressure (mmHg)	64 ± 7	60 ± 12	63 ± 10	57 ± 8	64 ± 9	57 ± 7
LVEDd (mm)	47.0 ± 3.0	48.7 ± 3.1	49.2 ± 3.4	48.5 ± 2.8	49.9 ± 3.0	50.0 ± 3.0
LVEDd/FFM (mm/kg)	1.28 ± 0.12	1.24 ± 0.12	1.24 ± 0.11	1.21 ± 0.13	1.24 ± 0.11	1.21 ± 0.12
LVEDd/BSA (mm/m^2^)	33.3 ± 2.3	33.6 ± 2.0	33.8 ± 2.1	33.3 ± 2.4	34.1 ± 2.4	33.7 ± 2.5
LV septal wall thicknesses (mm)	7.8 ± 0.6	8.4 ± 0.6	8.3 ± 0.7	8.3 ± 0.8	8.2 ± 0.8	8.5 ± 0.7
LV posterior wall thicknesses (mm)	8.1 ± 0.6	8.3 ± 0.7	8.5 ± 0.6	8.5 ± 0.9	8.5 ± 0.7	8.5 ± 0.8
LV mass (g)	122.6 ± 15.7	137.4 ± 16.6	141.8 ± 20.0	137.7 ± 13.3	143.8 ± 17.4	147.0 ± 20.1

FFM: fat-free mass, LVEDd: left ventricular end-diastolic dimension, BSA: body surface area. Occasion of measurement (T_measure_) is numbered from 0 (baseline) to 5 with a 1-unit increase representing 6 months.

### Performance change

**Fig 1:**
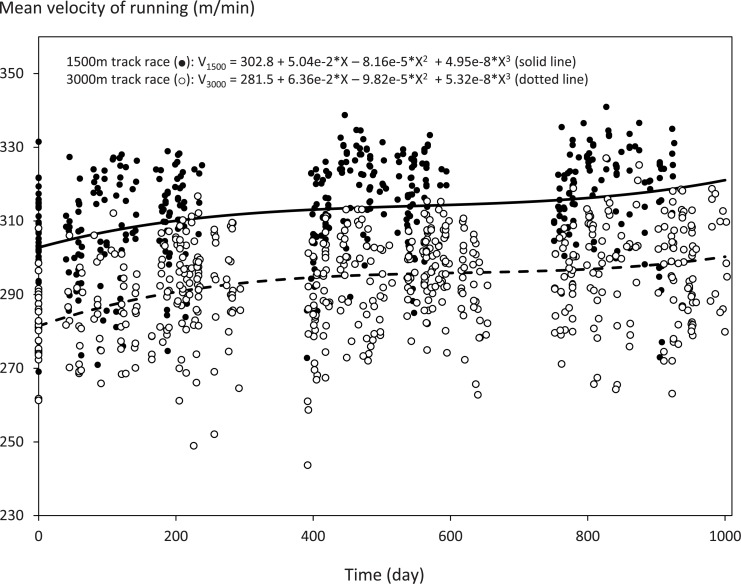
Changes in running velocities for 1500 m and 3000 m races. The cubic polynomial curves determined by multilevel analysis to best describe the trajectories of running velocities for 1500 m (V_1500_, solid line) and 3000 m (V_3000_, dotted line) track races over time among adolescent female distance runners. Closed and open circles represent the records of individual 1500 m and 3000 m races, respectively. X in the model equations denotes Time (day). All unadjusted estimates; *p* < 0.05 and *p* < 0.001, respectively.

Change in V_1500_ (m/min) and V_3000_ (m/min) were best described by cubic models (Fig 1). V_1500_ and V_3000_ consistently improved over 3 years of physical training.

### Modeling of LV geometrical change

According to unconditional mean model analyses, ICCs of LVEDd, LV septal and posterior wall thicknesses, and LV mass were 0.591, 0.304, 0.431, and 0.466, respectively. There was significant variance to be explained within subjects (Wald Z = 6.855, 7.067, 7.053, and 6.891, respectively, all *p* < 0.001), and the intercepts varied significantly across subjects (Wald Z = 3.432, *p* < 0.001; 2.685, *p* < 0.01; 3.223, *p* < 0.005; and 3.117, *p* < 0.005, respectively).

**Fig 2:**
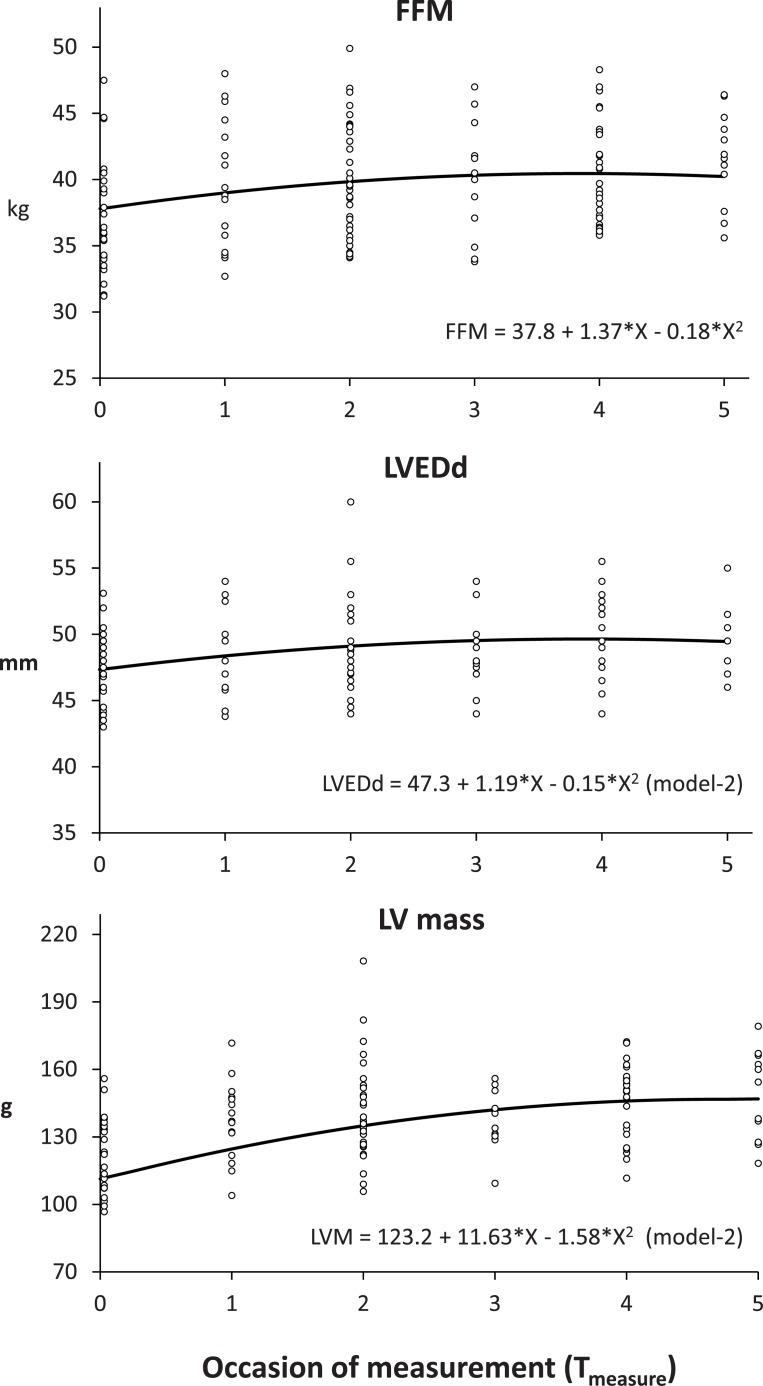
The trajectories of changes in fat-free mass (FFM), left ventricular end-diastolic dimension (LVEDd), and left ventricular mass (LV mass) among adolescent female distance runners. Occasion of measurement (T_measure_) is numbered from 0 (baseline) with a 1-unit increase representing 6 months. Open circles represent individual data. X in the polynomials denotes T_measure_.

Multilevel analysis demonstrated that quadratic models were significantly better than linear models to predict LVEDd and LV mass (see S1 Table). The increases in LVEDd and LV mass were initially rapid and subsequently slowed over time (Fig 2).

Changes in FFM and fat mass were best described by a quadratic model: FFM = 37.8 + (1.37 * T_measure_)–(0.18 * T_measure_^2^) and fat mass = 7.41 –(0.70 * T_measure_) + (0.13 * T_measure_^2^) (all unadjusted estimates; *p* < 0.001 and *p* < 0.01, respectively), indicating that the increase in FFM and decrease in fat mass were initially rapid and subsequently slowed over time. The coefficients of the quadratic model of FFM were similar to those of LVEDd, and thus similar trajectories were obtained in LVEDd and FFM (Fig 2). LVEDd or LVM were not significantly related to VO_2_max. (*p* > 0.05). Change in VO_2_max was best described by the FFM-adjusted model as follows: VO_2_max = 608.9 + (26.1 * T_measure_) + (54.2 * FFM) (all unadjusted estimates; *p* < 0.05).

Multilevel analysis demonstrated that FFM was significantly related to LVEDd and LV mass in both linear and quadratic models (see S2 Table). However, once LVEDd was adjusted for FFM, time components (T_measure_) lost their significance to predict LVEDd by any of the linear or quadratic models used. By contrast, training duration was significantly related to LV mass increase over time. An FFM-adjusted quadratic model significantly better predicted the change of LV mass than an FFM-adjusted linear model. FFM was not significantly related to LV septal or posterior wall thickness in either the linear or quadratic model. Since the coefficients for time components in these models were very small, the extent of changes in LV wall thicknesses were minimal (see S2 Table).

Table 2 shows the comparison between models to predict LVEDd and LV mass with or without FFM as a covariate. Considering FFM as a covariate significantly improved both the linear and quadratic models used to predict both variables.

**Table 2:** Comparison between models to predict the left ventricular (LV) end-diastolic dimension and LV mass with or without fat-free mass (FFM) as a covariate.

		FFM as a covariate	*–2LL*	Number of parameters	Change in –*2LL* for *χ* ^2^ test (*df*, N)
**LV end-diastolic dimension**					
linear models	model-1	–	603.628	5	
	model-3	+	582.714	6	20.914 * (*df* = 1, N = 131)
quadratic models	model-2	–	599.077	6	
	model-4	+	581.285	7	17.792 * (*df* = 1, N = 131)
**LV mass**					
linear models	model-1	–	1082.599	5	
	model-3	+	1062.175	6	20.424 * (*df* = 1, N = 131)
quadratic models	model-2	–	1070.193	6	
	model-4	+	1054.456	7	15.737 * (*df* = 1, N = 131)

* *p* < 0.01,

*–2LL*: minus twice the log-likelihood, N: total number of measurements.

Chi-square (*χ* ^2^) statistics were calculated by subtracting the –*2LL* of model-3 from that of model-1 and by subtracting the –*2LL* of model-4 from that of model-2 for linear and quadratic model comparisons, respectively. Degrees of freedom (*df*) for *χ* ^2^ tests were calculated by subtracting the number of parameters in model-1 from that in model-3 and by subtracting the number of parameters in model-2 from that in model-4 for comparison of quadratic models comparisons.

## Discussion

The homogeneity of the subjects’ background was a strength of the present study to assess the longitudinal changes in LV dimensions elicited by continuing exercise training. The training regimen was consistency provided by the same 2 coaches, and the volume, intensity, composition, and seasonal variation of training were not substantially changed during the study period. Runners of the same team usually train not individually but by running in a pack in Japanese high schools. Therefore, the training stimulus was assumed to be relatively similar among the subjects.

Another unique feature of our study is that it focused on modeling the trajectories of LV dimensions by repeating echocardiography over time. Care should be taken to the fact that the assumption of independence of error is violated in studies of repeated measures on individuals over time. In fact, ICCs of LV dimension variables were all high in our study, meaning that the assumption of independence of errors was likely violated. If traditional statistical methods such as repeated-measures ANOVA had been used to analyze such data, the chance of type I error would be inflated [17]. Therefore, we used multilevel analysis to demonstrate the changes in running performance as well as LV dimensions. To our knowledge, this study is the first to attempt longitudinal modeling of the trajectories of LV dimensions along with continuing intensive exercise training among endurance athletes using multilevel analysis.

It is important to characterize the stimulus of physical training to evaluate training-induced remodeling of the left ventricle. Performance of athletes (achievements or records) has been used often for that purpose [14][15][25] because it is difficult to control the quantity of volume and intensity of exercise training in a long-term observational study. In fact, Legaz-Arrese et al. [25] reported a relationship between LVEDd and running performance in a cross-sectional study. They also suggested change of LVEDd was associated with that of running performance in a longitudinal study [26]. The subjects of the present study were under chronic physical stress for the 3-year period. They were recruited to a nationally famous high school distance running team based on the promise of their former achievements and were highly motivated to succeed in track and field athletics and road races. Indeed, the velocity of official 1500 m and 3000 m races progressively increased over the study period, and VO_2_max increased even adjusted for FFM change. Accordingly, resting HR decreased. These observations were assumed to be indirect evidence for the subjects being exposed to incessant training stimulus. We characterized performance change by using growth curve analyses, and performance was nonlinearly but steadily increased. Furthermore, FFM and fat mass were increased and decreased, respectively. These trajectories were best described by a quadratic polynomial where the initial changes were manifested. Girls gain more fat than lean tissue at puberty in normal growth [27], but the opposite trends in body composition were observed in our subjects along with enhanced running performance. This finding indicates that the subjects had been engaging in strenuous physical exercise to such an extent that substantial development of the skeletal muscles along with combustion of body fat occurred coincident to the improvement in running speed, particularly while they were freshmen. This observation is in line with the fact that the biggest change in subjects’ physical stress occurred during their first year on the high school team, when the volume, intensity, and quality of training were amplified in comparison to those in their junior high school.

The left ventricles of our subjects were enlarged at baseline. Sharma et al. [4] reported LV dimensions of a large number of elite junior athletes in the UK (age 15.7 ± 1.4 years, not including distance runners). The LVEDd of our subjects at baseline was comparable to that of elite female junior athletes in the UK (47.0 ± 3.0 vs. 47.7 ± 3.3 mm, respectively). However, when mean LVEDd/BSA was calculated, our subjects had larger LVEDd/BSA than UK elite female junior athletes (33.3 vs. 28.9 mm/m^2^). The LVEDd/BSA of our subjects was also larger than that of adolescent elite African male soccer players (33.3 ± 2.3 vs. 28.3 ± 2.3 mm/m^2^) [28]. Furthermore, the ratio of LV dimension to BSA in our subjects was comparable to those of adult Italian elite female cyclists (LVEDd/BSA: 31.7 mm/m^2^) and cross-country skiers (LVEDd/BSA: 31.9 mm/m^2^) [6] and even French professional cyclists (LVEDd/BSA: 31.9 ± 2.2 mm/m^2^) [14]. All our subjects had previous careers of intensive endurance training in junior high school before entry into the study, which implies that the response of LV adaptation to exercise training had already started. The impact of exercise training on LVEDd is most prominent in endurance athletes [1][2][5][6]. The exercise training our subjects had engaged in was purely of endurance type, which would enhance their LV dilatation from the early stage of life. However, it is equally likely that such characteristics could reflect genetic pre-selection. That is, those female runners with genetically determined large left ventricles would be expected to perform better in endurance events.

The longitudinal changes in LVEDd and LV mass were best described by quadratic polynomials of training duration (T_measure_), which indicated that the left ventricle was rapidly enlarged at the initial phase of the training career of subjects in the high school team (Fig 2). As the quantity and quality of training were substantially amplified in the first year among subjects in the high school team, it may not be coincidental that relatively large increases in these LV dimensions were observed initially. In the context of the theory of “athlete’s heart,” in which the left ventricle enlarges in response to sustained physical stress, it seems rational to assume that the substantial increase in physical stress in the first year compelled the relatively large adaptive change in the LV dimensions. Nevertheless, in the models for LVEDd adjusted for FFM, time components lost their significance to predict LVEDd. Indeed, we observed similar coefficients of time components in the quadratic models of LVEDd and FFM and, accordingly, the collateral trends in the trajectories of both variables (Fig 2). These findings suggested that the variance of change in LVEDd among our subjects could be explained by the change in FFM when the time trend analysis comprises a chronic stage of adaptation with exercise training. A similar finding was reported by Pelliccia et al. [15] who assessed LV dimensions of serial participants in endurance disciplines at the Olympic Games over many years. Unfortunately, FFM values of the subjects were not available in their study; however, LVEDd of the subjects did not change with long-term intensive exercise training while BSA remained unchanged. Still, the results of our study do not rule out the potential influence of exercise training on long-term LV enlargement. FFM increases in accordance with an increase in physical stress owing to exercise training. Therefore, changes in the amount or intensity of training could be confounders in the apparent association between the changes in LVEDd and FFM. Moreover, the duration of training does not quantitatively represent the extent of physical stress owing to exercise training. Thus, it is possible that the impact of FFM would surpass that of training duration when using FFM and training duration as covariates to explain the changes in LV dimensions among adolescent female runners, considering the chronic stage of LV morphological adaptation. Nevertheless, it is difficult to quantify the physical stress of athletes, and duration of the training career is often used in clinical practice to estimate the long-term effect of exercise training. Therefore, to use training duration for the analysis of long-term LV morphological change is a rational approach among athletes.

Previous cross-sectional studies indicated that LVEDd, LV end-diastolic volume, and total heart volume were independent predictors of VO_2_max [29][30][31]. However, the present study found that the change in LVEDd was not a significant predictor for the change in VO_2_max. We believe that our finding is not necessarily inconsistent with the findings of the previous studies. Owing to the highly homogeneous backgrounds of our subjects, the variability in VO_2_max was lower in our subjects than in those from the previous studies, which used heterogeneous samples, including sedentary subjects and patients. When the variability of a dependent variable is small in the analyses, a statistical significance of covariates would not be obtained. Furthermore, we observed longitudinal changes in VO_2_max and LV dimensions and found no statistical association between them. The absence of an association between changes in VO_2_max and LVEDd does not necessarily indicate the absence of an association between VO_2_max and LVEDd at a certain time-point. As LVEDd adjusted for FFM did not change in our subjects and LVEDd was not a significant predictor for the change in VO_2_max, it appears that central adaptation to exercise training did not progress further with continuation of exercise training among the subjects. Nevertheless, running performance or VO_2_max improved during the training period. This could be explained by factors other than central adaptation, such as improvement in the running economy and oxygen utilization by the skeletal muscles.

FFM was a significant predictor for LV mass. This finding is consistent with the results of a previous report by D'Ascenzi et al. [32], in which LV dimensions and FFM were followed in 23 male top-level athletes at pre-season, mid-season, and after detraining. They conducted pre- and post-test-type comparisons using the difference in LV dimensions and FFM and found that change in LV mass was closely associated with change in FFM. In the present study, LV mass significantly increased even after adjustment for FFM. Similarly, Pressler et al. [33] found that LV mass was significantly higher in athletes than in sedentary controls even after adjustment for FFM. The reason was not clear, but they suggested that training-induced adaptations beyond FFM might occur in LV muscle.

Although FFM was a better predictor of the LV cavity dimension than BSA [33][34], many studies of “athlete’s heart” have used BSA or its allometric scale as a covariate for the adjustment of LV dimensions [3][5][9][10]. However, caution must be taken when using BSA for athletes, particularly to assess longitudinal change. BSA would not change if height and weight do not change over time, and thus would not yield any information about body composition. It is plausible among endurance athletes that FFM increases to the extent that fat mass decreases, while there is no substantial change over time in weight or, consequently, BSA. In this situation, LVEDd would increase in accordance with the FFM increase, and thus LVEDd/BSA would increase. This change should be explained by the change of FFM but might be spuriously interpreted as a direct consequence of continued intensive exercise training [34]. This would be the case when LV dimensions are indexed for height raised to an exponential power of 2.7, which has been used often for the adjustment. Thus, it is crucially important to use FFM to assess the longitudinal change in LV morphology among athletes who continue exercise training. In clinical settings, many practitioners might be interested in the unadjusted value of LVEDd. However, the predictive equation of LVEDd involving only FFM as a variable provided in the present study (S2 Table, model 4) would be useful when the chronic stage of LV adaptation to long-term physical stress is assessed among adolescent female runners.

One of the limitations of the present study was not considering stages of maturation, which might have varied among the adolescent subjects. Normal growth and development could potentially influence LV dimensions. Since no non-training control group was analyzed in our study, the extent that normal growth can account for the findings is uncertain. Secondly, the number of subjects was relatively small for a multilevel analysis using maximum likelihood estimation methods [24]. However, a simulation study indicated that the estimates of the regression coefficients in multilevel analysis for relatively simple models are unbiased even if the sample is as small as 10 groups of 5 units [35]. It is challenging to increase the number of subjects while maintaining the strict homogeneity achieved in the present study as well as to conduct a prospective, longitudinal, multi-year study on a large number of subjects in terms of the cost involved.

The marked homogeneity of the subjects is a major strength of this study, but for this very reason, the results should also be carefully applied to athletes as a whole or athletes of other backgrounds. Particularly, the extent of physical stress, i.e., the volume, intensity, and duration of training, among our subjects was relatively limited in comparison to those of adult elite athletes. A different scenario for the consequences of chronic exercise training regarding LV remodeling is expected among these athletes. The same study design should be used in male athletes, athletes who engage in other disciplines, and athletes at different stages of life or different levels of achievement.

## Conclusions

Multilevel analysis of long-term LV adaptation to exercise training indicated that the development of FFM was an important factor to determining LV dimensions in female adolescent distance runners with prior training. When FFM was adjusted, further LV cavity enlargement was not observed even with enhanced running performance and VO_2_max increase along with continued endurance training among these athletes.

## Supporting Information

S1 Table Multilevel analyses of linear and quadratic models to predict changes in left ventricular (LV) dimensions (model-1 and -2) with chi-square (*χ* ^2^) tests for model comparison. (PDF)

S2 Table Multilevel analyses of linear and quadratic models adjusted for fat-free mass (FFM) to predict changes in left ventricular (LV) dimensions (model-3 and -4) with chi-square (*χ* ^2^) tests for model comparison. (PDF)
