# Supplementary material for: Modeling of Longitudinal Changes in Left Ventricular Dimensions among Female Adolescent Runners
Source: PLoS One. 2015 Oct 15;10(10):e0140573. doi: 10.1371/journal.pone.0140573 (PMC4607476; doi:10.1371/journal.pone.0140573)
Supplement: S1 Table — (PDF) [file pone.0140573.s001.pdf]

S1 Table. Multilevel analyses of linear and quadratic models to predict changes in left ventricular (LV) dimensions (model-1 and -2) with chi-square (χ^2) tests for model comparison.

	b	SE_b	95% CI	Type III test	*–2LL* (parameters)	Change in *–2LL* (model 1 vs. 2) for χ^2 test (*df*, N)
**LV end-diastolic dimension**		**: model-1 (linear)**				
intercept	47.719	0.518	46.682, 48.758	$F(1, 57.264) = 8477.130$ *	603.628	
$T_{measure}$	0.486	0.122	0.240, 0.731	$F(1, 47.365) = 15.834$ *	(5)	
		**: model-2 (quadratic)**				
intercept	47.339	0.544	46.252, 48.425	$F(1, 70.652) = 7550.185$ *		
$T_{measure}$	1.186	0.346	0.497, 1.874	$F(1, 87.270) = 11.722$ *	599.077	4.551 †
$T_{measure}^2$	-0.152	0.070	-0.292, -0.012	$F(1, 87.649) = 4.679$ †	(6)	(*df* = 1, N = 131)
**LV septal wall thickness**		**: model-1 (linear)**				
intercept	7.950	0.121	7.708, 8.190	$F(1, 79.667) = 4303.597$ *	267.767	
$T_{measure}$	0.115	0.039	0.038, 0.194	$F(1, 49.884) = 8.923$ †	(5)	
		**: model-2 (quadratic)**				
intercept	7.856	0.132	7.595, 8.117	$F(1, 107.111) = 3558.290$ *		
$T_{measure}$	0.300	0.109	0.082, 0.518	$F(1, 89.451) = 7.509$ ¶	264.637	3.130
$T_{measure}^2$	-0.041	0.022	-0.085, 0.003	$F(1, 89.786) = 3.374$	(6)	(*df* = 1, N = 131)
**LV posterior wall thickness**		**: model-1 (linear)**				
intercept	8.178	0.110	7.958, 8.398	$F(1, 70.902) = 5488.720$ *	223.685	
$T_{measure}$	0.085	0.033	0.018, 0.152	$F(1, 39.610) = 6.638$ †	(5)	
		**: model-2 (quadratic)**				
intercept	8.081	0.117	7.848, 8.313	$F(1, 94.336) = 4763.399$ *		
$T_{measure}$	0.277	0.089	0.100, 0.453	$F(1, 92.178) = 9.651$ §	218.534	5.151 †
$T_{measure}^2$	-0.042	0.018	-0.077, -0.006	$F(1, 95.387) = 5.313$ †	(6)	(*df* = 1, N = 131)
**LV mass**		**: model-1 (linear)**				
intercept	127.219	2.991	121.241, 133.196	$F(1, 62.823) = 1809.249$ *	1082.599	
$T_{measure}$	4.383	0.781	2.803, 5.964	$F(1, 38.416) = 31.504$ *	(5)	
		**: model-2 (quadratic)**				
intercept	123.225	3.123	117.008, 129.443	$F(1, 77.808) = 1556.850$ *		
$T_{measure}$	11.632	2.128	7.401, 15.863	$F(1, 79.410) = 29.941$ *	1070.193	12.406 ¶
$T_{measure}^2$	-1.575	0.432	-2.435, -0.715	$F(1, 77.536) = 13.300$ *	(6)	(*df* = 1, N = 131)

* $p < 0.001$, § $p < 0.005$, ¶ $p < 0.01$, † $p < 0.05$. N: total number of measurements, b: unadjusted regression coefficient, SEb: standard error of coefficient, CI: confidence interval, *–2LL*: minus twice the log-likelihood. Occasion of measurement ($T_{measure}$) is numbered from 0 (baseline) to 5 with a 1-unit increase representing 6 months. All parameter estimates are unstandardized regression coefficients.

χ^2 statistics were calculated by subtracting the *–2LL* of model-2 from that of model-1. Degrees of freedom (*df*) for χ^2 tests were calculated by subtracting the number of parameters in model-1 from that in model-2.
