# Supplementary material for: Modeling of Longitudinal Changes in Left Ventricular Dimensions among Female Adolescent Runners
Source: PLoS One. 2015 Oct 15;10(10):e0140573. doi: 10.1371/journal.pone.0140573 (PMC4607476; doi:10.1371/journal.pone.0140573)
Supplement: S2 Table — (PDF) [file pone.0140573.s002.pdf]

S2 Table. Multilevel analyses of linear and quadratic models adjusted for fat free mass (FFM) to predict changes in left ventricular (LV) dimensions (model-3 and -4) with chi-square (χ^2) tests for model comparison.

	b	SE_b	95% CI	Type III test	*-2LL* (parameters)	Change in *–2LL* (model 3 vs. 4) for χ^2 test (*df*, N)
**LV end-diastolic dimension**	**: model-3 (linear)**					
intercept	31.738	3.377	24.986, 38.490	$F(1, 61.384) = 88.327$ *		
$T_{measure}$	0.241	0.125	-0.010, 0.491	$F(1, 60.820) = 3.689$	582.714	
FFM	0.419	0.088	0.243, 0.594	$F(1, 62.075) = 22.821$ *	(6)	
	**: model-4 (quadratic)**					
intercept	32.487	3.411	25.657, 39.317	$F(1, 56.847) = 90.729$ *		
$T_{measure}$	0.636	0.353	-0.063, 1.336	$F(1, 100.398) = 3.256$		
$T_{measure}^2$	-0.083	0.069	-0.219, 0.054	$F(1, 92.694) = 1.447$	581.285	1.429
FFM	0.394	0.089	0.214, 0.573	$F(1, 57.278) = 19.348$ *	(7)	(*df* = 1, N = 131)
**LV septal wall thickness**	**: model-3 (linear)**					
intercept	7.191	0.800	5.585, 8.800	$F(1, 49.530) = 80.865$ *		
$T_{measure}$	0.103	0.041	0.021, 0.184	$F(1, 55.358) = 6.338$ †	266.856	
FFM	0.020	0.021	-0.022, 0.062	$F(1, 50.016) = 0.919$	(6)	
	**: model-4 (quadratic)**					
intercept	7.317	0.804	5.701, 8.933	$F(1, 48.295) = 82.868$ *		
$T_{measure}$	0.279	0.114	0.053, 0.504	$F(1, 96.804) = 6.011$ †		
$T_{measure}^2$	-0.038	0.023	-0.083, 0.007	$F(1, 93.079) = 2.873$	264.177	2.679
FFM	0.014	0.021	-0.028, 0.057	$F(1, 48.389) = 0.462$	(7)	(*df* = 1, N = 131)
**LV posterior wall thickness**	**: model-3 (linear)**					
intercept	7.489	0.753	5.980, 8.998	$F(1, 58.178) = 98.846$ *		
$T_{measure}$	0.074	0.035	0.003, 0.145	$F(1, 45.732) = 4.409$ †	222.836	
FFM	0.018	0.020	-0.021, 0.057	$F(1, 56.727) = 0.853$	(6)	
	**: model-4 (quadratic)**					
intercept	7.709	0.758	6.188, 9.229	$F(1, 54.016) = 103.359$ *		
$T_{measure}$	0.262	0.094	0.076, 0.448	$F(1, 101.675) = 7.828$ ¶		
$T_{measure}^2$	-0.040	0.018	-0.076, -0.003	$F(1, 100.038) = 4.669$ †	218.289	4.547 †
FFM	0.010	0.020	-0.030, 0.050	$F(1, 54.317) = 0.246$	(7)	(*df* = 1, N = 131)
**LV mass**	**: model-3 (linear)**					
intercept	35.073	19.767	-4.559, 74.705	$F(1, 53.880) = 3.148$		
$T_{measure}$	2.956	0.794	1.358, 4.554	$F(1, 45.730) = 13.863$ *	1062.175	
FFM	2.415	0.513	1.386, 3.444	$F(1, 54.412) = 22.132$ *	(6)	
	**: model-4 (quadratic)**					
intercept	43.260	19.639	3.870, 82.650	$F(1, 53.066) = 4.852$ †		
$T_{measure}$	8.674	2.167	4.369, 12.979	$F(1, 90.849) = 16.017$ *		
$T_{measure}^2$	-1.204	0.425	-2.049, -0.359	$F(1, 83.357) = 8.029$ ¶	1054.456	7.719 ¶
FFM	2.120	0.515	1.087, 3.153	$F(1, 53.330) = 16.929$ *	(7)	(*df* = 1, N = 131)

* $p < 0.001$, § $p < 0.005$, ¶ $p < 0.01$, † $p < 0.05$. N: total number of measurement, *b*: unadjusted regression coefficient, SE_b: standard error of coefficient, CI: confidence interval, *–2LL*: minus twice the log-likelihood. Occasion of measurement ($T_{measure}$) is numbered from 0 (baseline) to 5 with a 1-unit increase representing 6 months. All parameter estimates are unstandardized regression coefficients.

χ^2 statistics were calculated by subtracting the *–2LL* of model-4 from that of model-3. Degrees of freedom (*df*) for χ^2 tests were calculated by subtracting the number of parameter in model-3 from that in model-4.
